# Fucosyltransferase 4 and 7 mediates adhesion of non-small cell lung cancer cells to brain-derived endothelial cells and results in modification of the blood–brain-barrier: in vitro investigation of CD15 and CD15s in lung-to-brain metastasis

**DOI:** 10.1007/s11060-019-03188-x

**Published:** 2019-05-18

**Authors:** Samah A. Jassam, Zaynah Maherally, Keyoumars Ashkan, Geoffrey J. Pilkington, Helen L. Fillmore

**Affiliations:** 1 Cellular and Molecular Neuro-Oncology Research Group, School of Pharmacy and Biomedical Sciences, University of Portsmouth, White Swan Road, Portsmouth, P01 2DT UK; 2 Neuro-Surgery, King’s College Hospital, Denmark Hill, London, SE5 9RS UK

**Keywords:** CD15, CD15s, NSCLC, Brain metastasis, FUT4, FUT7

## Abstract

**Purpose:**

Metastatic non-small cell lung (NSCLC) cancer represents one of the most common types of brain metastasis. The mechanisms involved in how circulating cancer cells transmigrate into brain parenchyma are not fully understood. The aim of this work was to investigate the role of fucosylated carbohydrate epitopes CD15 and sialyated CD15s in cancer adhesion to brain-derived endothelial cells and determine their influence in blood–brain barrier (BBB) disruption

**Methods:**

Three distinct, independent methods were used to measure brain endothelial integrity and include voltohmmeter (EVOM™), impedance spectroscopy (CellZscope®) and electric cell-substrate impedance sensing system (ECIS™). Two fucosyltransferases (FUT4 and 7) responsible for CD15 and CD15s synthesis were modulated in four human cancer cell lines (three lung cancer and one glioma).

**Results:**

Overexpression of CD15 or CD15s epitopes led to increase in adhesion of cancer cells to cerebral endothelial cells compared with wild-type and cells with silenced CD15 or CD15s (p < 0.01). This overexpression led to the disruption of cerebral endothelial cell monolayers (p < 0.01). Knockdown of *FUT4* and *FUT7* in metastatic cancer cells prevented disruption of an in vitro BBB model. Surprisingly, although the cells characterised as ‘non-metastatic’, they became ‘metastatic’ -like when cells were forced to over-express either *FUT4 or FUT7*.

**Conclusions:**

Results from these studies suggest that overexpression of CD15 and CD15s could potentiate the transmigration of circulating NSCLC cells into the brain. The clinical significance of these studies includes the possible use of these epitopes as biomarkers for metastasis.

**Electronic supplementary material:**

The online version of this article (10.1007/s11060-019-03188-x) contains supplementary material, which is available to authorized users.

## Introduction

The majority of intracranial tumours are secondary metastases originating from primary non-central nervous system (non-CNS) cancers. 20–40% of patients with systemic cancers develop secondary brain tumours [1, 2]. The highest incidence of brain metastasis is seen in lung cancer patients (40–50%) followed by breast (20–30%) and melanoma (5–10%) [1, 3, 4]. As overall survival in patients with primary non-CNS cancers has improved, the incidence of metastasis to the brain has also increased, possibly due to circulating cancer cells which persist despite patient remission. The central nervous system (CNS) is considered a site of refuge as cancers are protected from most systemic therapies by the blood–brain barrier (BBB). The BBB is a dynamic and selective defensive barrier, which maintains a highly specific environment within the CNS by inhibiting both fluctuations of plasma components and entry of substances that may potentially cause brain toxicity [5]. Brain vascular endothelial cells are specific to the BBB with low pinocytosis and no fenestrations [6] and these cells may play a crucial role in the homing process of brain metastasis from breast cancer [7] and melanoma [8]. Although, leukocyte-like mechanisms have been suggested in the extravasation process of some cancer cells [9, 10], the transmigration of cancer cells particularly to the brain has not been thoroughly investigated. Recently, we have characterised CD15 and CD15s (sialyl CD15) expression in non-small cell lung cancer cells and their potential importance in adhering to brain derived endothelial cells using a model that mimics physiological blood flow [11, 12]. CD15 and CD15s, also known as Lewis^x^ and sialyl Lewis^x^ respectively, are classified as Type II Lewis antigens and are synthesized by specific fucosyltransferases [13]. To gain a better understanding on the role of CD15 and CD15s in lung cancer metastasis to the brain, two fucosyltransferases, FUT4 and 7, responsible for their corresponding synthesis were genetically modulated in non-small cell lung cancer cells. The effect of these genetically modified cells on endothelial cell adhesion and on integrity of an in vitro BBB model was then evaluated.

## Materials and methods

### Cell culture

The human cerebral microvascular endothelial cell line (hCMEC/D3) was donated by Professor Couraud (Institute of Cochin, INSERM, Paris, France) [14] and cultured in endothelial basal medium-2 (EGM-2) (Lonza, Germany) supplemented with 2% human serum (Sigma, UK). Primary NSCLC cells (COR-L105) were purchased from Sigma, UK; metastatic NSCLC cells from cervical lymph node (NCI-H1299) from ATCC, UK and low-passage biopsy-derived brain-metastatic NSCLC cells, obtained from a patient with lung-brain metastatic cancer (SEBTA-001) as well as a biopsy-derived primary glioblastoma (GBM) cell line (UP-007) both established “*in house”*. Cell lines were maintained at 5% CO_2_ and in a humidified atmosphere at 37 °C. All lines were subjected to routine mycoplasma testing, and cell authentication [15].

### Antibodies

Mouse monoclonal anti-CD15 (MEM-158) (Santa Cruz Biotechnology, USA) was used at the following dilutions: 1:100 for immunocytochemistry (ICC) and 1:10 for flow cytometry (FC). Mouse monoclonal anti-sialyl CD15 (BD Biosciences, UK) was used at the following dilutions: 1:50 for ICC and 1:25 for FC. Secondary antibodies, fluorochrome-conjugated Alexa Fluor-488 and 568 IgM (Thermo Fisher Scientific, UK) were used for ICC and FC at 1:500.

### Overexpression and knockdown of CD15/FUT4 and CD15s/FUT7

Overexpression of CD15 and CD15s was carried out by transfecting cell lines: SEBTA-001, NCI-H1299, COR-L105 and UP-007 with human non-viral cDNA containing a unique construct of alpha (1, 3) fucosyltransferase (*FUT4*): (OriGene, USA, *FUT4* (NM_002033) or alpha (1, 3) fucosyltransferase (*FUT7*) (OriGene, USA, *FUT7* (NM_004479). cDNA constructs contained a GFP expression cassette. Transfection was carried out by using TurboFectin8.0 as per the manufacturer’s protocol (OriGene, USA). In parallel, endogenous expression of CD15/*FUT4* and CD15s/*FUT7* were knocked down using four different human-*FUT4* and *FUT7* unique 29 mer shRNA constructs in pGF-V-RS GFP vectors (OriGene, USA).

### Immunocytochemistry

Cells were seeded onto sterile coverslips at 1 × 10^3^/well overnight, fixed with 4% paraformaldehyde (PFA) (Sigma, UK) followed by three washes with phosphate-buffered saline (PBS) (Sigma, UK). Non-specific antigens were blocked with 10% goat serum (Sigma, UK) then incubated with the primary antibody for 1 h followed by 30 min incubation with their respective secondary antibody (Thermo Fisher Scientific UK). Hoechst Blue (Cell Signalling Technology, UK) was used as nuclear counterstain. Coverslips were examined using a Zeiss Axio fluorescence microscope and images were captured using a Volocity Image Analysis Software (V 5.2, Perkin Elmer).

### Confocal microscopy

Images were obtained from a Zeiss LSM 510 Meta Axioskop2 confocal microscope (× 40 and × 100 objectives) using lasers with excitation wavelengths of 405 nm (blue), 488 nm (green) and 568 nm (red) and with diode, argon and HeNe1 lasers respectively. Identical settings were used to image negative controls in which primary antibody was replaced with non-specific Isotype.

### Flow cytometry analysis

Cells were collected via gentle scraping, blocked in 2% goat serum/PBS (Sigma, UK) and primary antibodies applied while non-specific IgM isotype was added to the negative control and incubated for 30 min. Cells were then washed and secondary antibodies (ThermoFisher Scientific, UK) applied for 15 min followed by more washes before transferring to fluorescence-activated cell sorting (FACS) tubes (BD Biosciences, UK) containing 5μL of Propidium iodide (PI) (Cell Signalling Technology, UK). Samples were analysed using a 4-color-multiparameter FACS Calibur (BD Biosciences-UK). Each experiment was repeated three independent times in triplicate. Data were represented as percentage of positive cell population.

### Adhesion assay

An adhesion assay kit, CytoSelect Tumor-Endothelium (Cell Biolabs, UK) was used [11, 12]. Briefly, 1 × 10^6^ brain endothelial cells/well were seeded onto a sterile surface coated with fibronectin (10 mg/mL). Cells were grown to form a complete monolayer. Cancer cells, labelled with a green fluorescent dye (Cell Biolabs, UK), were seeded on surface of the activated (with 25 pg/mL TNF-α) hCMEC/D3 monolayer and incubated for 90 min. Non-adherent cells were washed thoroughly with pre-warmed PBS. Representative adherent cells were assessed using a POLARstar OPTIMA microplate reader (BMG LABTECH, UK). The experiment was repeated 3 independent times in triplicate.

### Trans-endothelial migration studies

### Voltohmmeter (EVOM™)

Polycarbonate membrane Transwell inserts (24 well, 8.0 μm pore size) (Thermo Fisher, UK, UK) were pre-coated with 10 μg/mL human fibronectin (Sigma, UK) prior to addition of medium supplemented with TNF-α (25 pg/mL) and 1 × 10^5^ cells/well of hCMEC/D3 to apical side of the inserts. Readings were recorded using a voltohmmeter (EVOM™) (World Precision Instruments, USA). When resistance reached a plateau, 2.0 × 10^4^ cells/well were added on top of the hCMEC/D3 monolayer. Five readings were recorded per day and resistance measurement monitored for a further 5-day period and Ohm’s law applied.

#### Impedance spectroscopy (CellZscope®)

hCMEC/D3 cells (1 × 10^5^/well) were seeded on fibronectin-coated (10 µg/mL) polycarbonate Transwell inserts (24 well, 8.0 μm pore size) (Thermo Fisher, UK), placed in the CellZscope® module and incubated in a sterile, humidified, 37 °C and 5% CO_2_ incubator. Resistance values were recorded using an automated cell monitoring system, CellZscope® (nanoAnalytics, UK) until they reached a plateau wherein 2.0 × 10^4^ cells/well were added on top of the hCMEC/D3 monolayer. TER values, expressed in Ω · cm^2^, were recorded in real-time over a 5-day period post-addition of cancer cells. All experiments were carried out in triplicate and repeated at least three times.

#### Electric cell-substrate impedance sensing system (ECIS™)

ECIS arrays (8W10E+ , ibidi, Germany) were stabilised with l-cysteine (10 nM; 10 min incubation; Sigma, UK), washed in Hank’s balanced salt solution (Fisher, UK) and coated with 10 mg/mL fibronectin for 2 h followed by seeding of 7.5 × 10^4^ cells/chamber of hCMEC/D3 in media supplemented with TNF-α (25 pg/mL). Resistance was monitored using an ECIS Zθ (Applied Biophysics, USA) system until a peak resistance was achieved. 2.0 × 10^4^ of cancer cells were then added onto the endothelial cell monolayer. Resistance values were obtained in Ω. The confluency of the endothelial cell monolayer was also evaluated by using an Olympus 1X71 inverted phase contrast microscope.

### Statistical analysis

All experiments were performed three times in triplicate, and data expressed as + SE. Statistical analyses were performed using One-way ANOVA followed by Tukey’s multiple comparison post hoc tests using Graph Pad Prism 6 software for analysis.

## Results

**Fig. 1:**
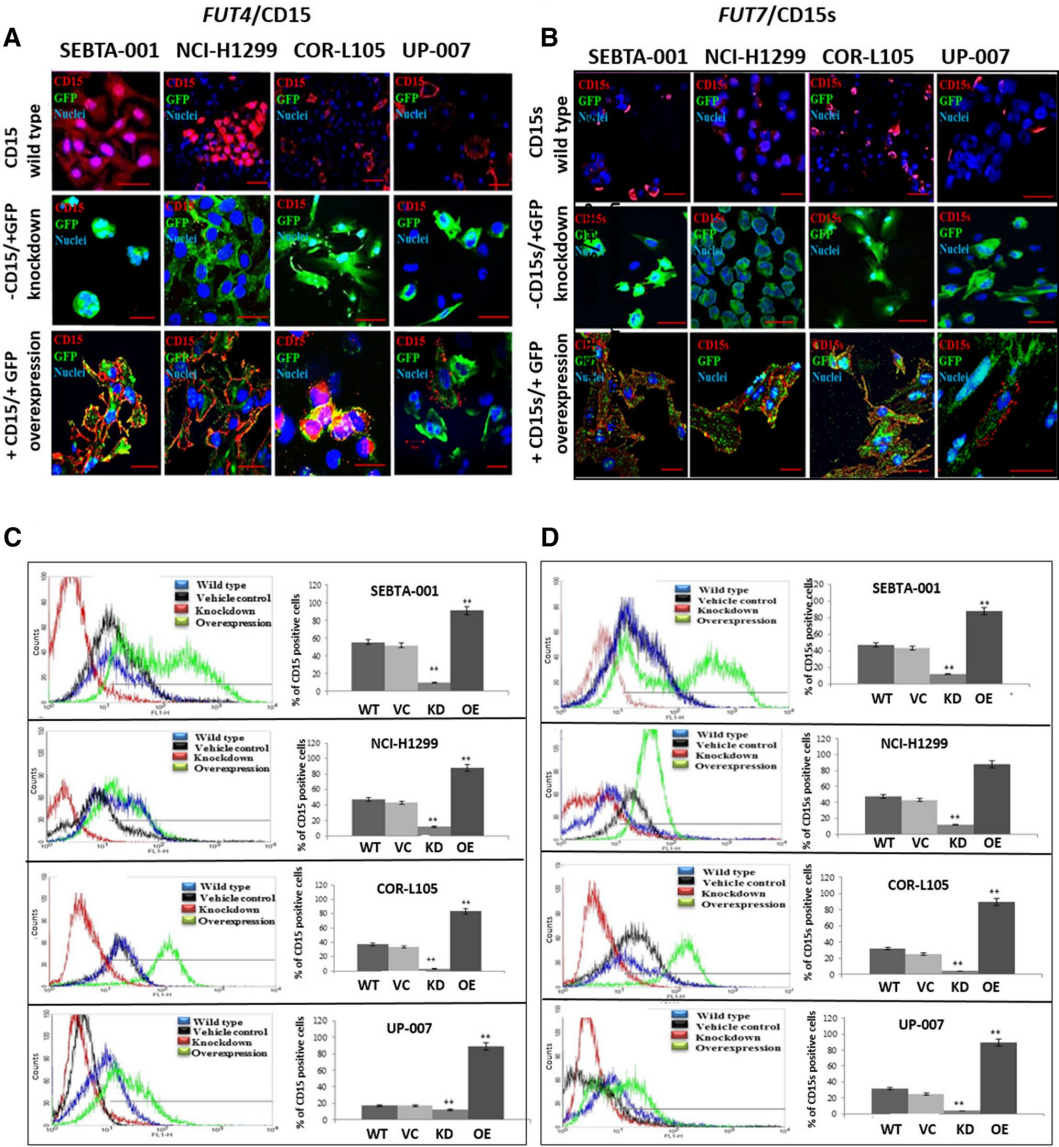
CD15 and CD15s expression in *FUT4/FUT7*-(cDNA and shRNA) transfected cells. Target cells (SEBTA-001, NCI-H1299, COR-L105 and UP-007) were transfected, either with plasmids containing *FUT4* or *FUT7*-cDNA to induce CD15 or CD15s overexpression, or with *FUT4* or *FUT7*-shRNA constructs for CD15 or CD15s knockdown. **a**, **c***FUT4*/CD15. **b**, **d***FUT7*/CD15s. **a**, **b** Representative confocal images showing localisation of **a** CD15 or **b** CD15s (red) cells grown on coverslips. **c**, **d** Representative FC analysis of CD15 or CD15s expression. The histograms show percentages of CD15/CD15s positive cells in wild type controls (blue) where no treatment applied, vehicle controls were transfected with non-coding plasmid (black), cells transfected with shRNA for *FUT4* or *FUT7* (red) and in cells transfection with cDNA for *FUT4* or *FUT7* (green). (**P < 0.01) difference compared to the wild type. N = 3. No treatment was applied to wild type controls and vehicle controls were transfected with non-coding plasmids. Scale bar = 20 μm

Three NSCLC cell lines and one GBM cell line (SEBTA-001, NCI-H1299, COR-L105 and UP-007) were transfected with either a full-length *FUT4-GFP* for CD15-overexpression or *FUT4* shRNA constructs for CD15-knockdown. CD15 protein expression was examined using ICC, and FC analyses (Fig. 1a, c). The highest levels of CD15 protein expression were seen on the surface of the NSCLC metastatic cell lines (NCI-H1299 and SEBTA-001), with lower cell surface expression in the non-metastatic cell lines (COR-L105 and UP-007), consistent with our previous report [11]. Expression of CD15 in cells transfected with *FUT*4 shRNA constructs demonstrated knockdown of CD15 expression (Fig. 1a, c). shRNA knockdown of *FUT4* resulted in a significant decrease in CD15 positive cells in SEBTA-001, NCI-H1299, COR-L105, and UP-007 cells (Fig. 1c; p < 0.01). In cell lines transfected with *FUT4* cDNA constructs, there was an increase in CD15 expression (Fig. 1a, c). In SEBTA-001 CD15 expression increased from 54.7 to 92%, in NCI-H1299 from 76 to 90%, in COR-L105 from 23 to 89% and in UP-007 from 13.4 to 84% (Fig. 1c; p < 0.01).

Using the same four cell lines and methods mentioned above, the effects of either over-expressing *FUT7* or silencing *FUT7* on CD15s expression was determined. The absence of CD15s was noted in cells treated with *FUT7-*shRNA (Fig. 1b, d). Expression of CD15s was significantly decreased post-transfection with *FUT7*-shRNA in all the cell lines: in SEBTA-001, NCI-H1299, COR-L105, and UP-007 (p < 0.01). FC analyses results showed a significant increase in CD15s expression on cells when transfected with *FUT7*-cDNA. The number of CD15s positive cells increased from 55 to 88% in SEBTA-001, 51–78% in NCI-H1299, 33–53.8% in COR-L105 and 17–45% in UP-007 (Fig. 1d, p < 0.01). These results suggest a strong correlation exists between genetic manipulation of *FUT4/FUT7* and CD15/CD15s expression.

**Fig. 2:**
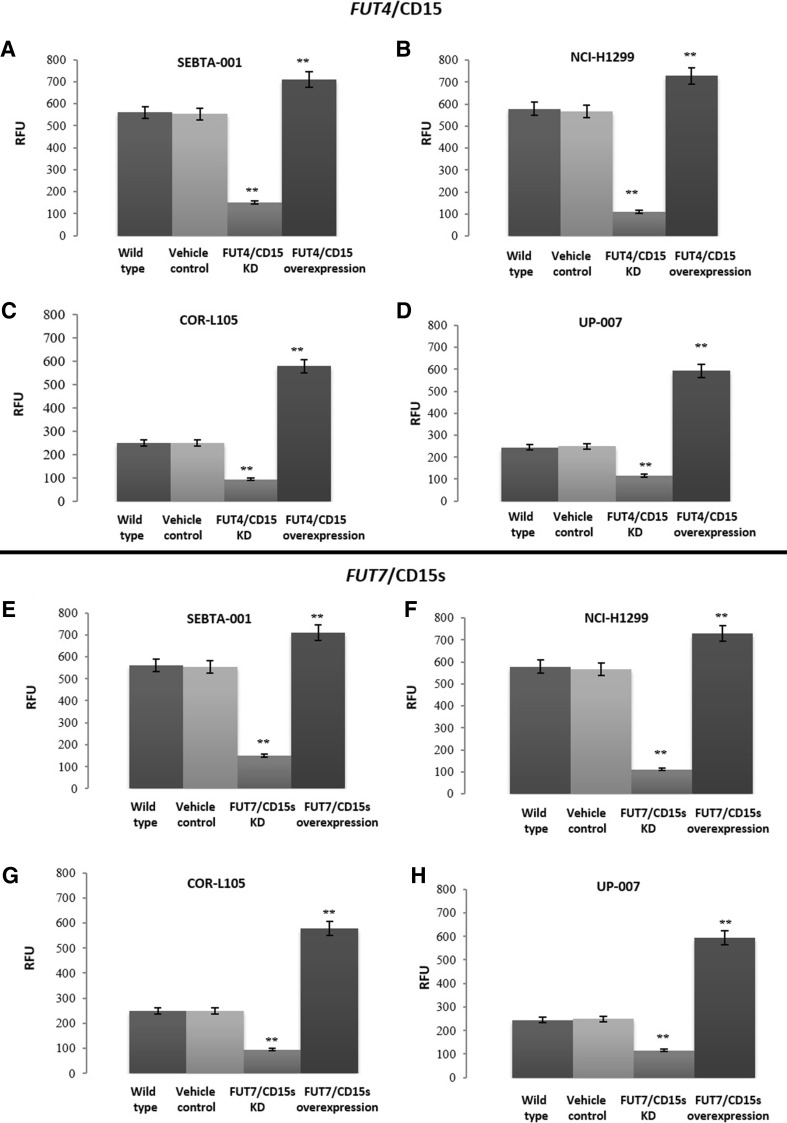
Adhesion of cancer cells following genetic manipulation of CD15 and CD15s. Adhesion of cancer cells following *FUT4*/CD15 and *FUT7*/CD15s manipulation on metastatic NSCLC SEBTA-001 (**a**, **e**), NCI-H1299 (**b**, **f**) and non-metastatic NSCLC: COR-L105 (**c**, **g**), UP-007 (**d**, **h**). Cancer cells were incubated for 90 min on a monolayer of activated brain endothelial cells (hCMEC/D3). Non-adherent cancer cells were washed off followed by lysis of cells and quantified via a microplate reader at 480–520 nm. (**P < 0.01), N = 3

Following genetic manipulation of *FUT4*/CD15 or *FUT7*/CD15s, changes in cell adhesion to an endothelial cell monolayer hCMEC/D3 was investigated. SEBTA-001 and NCI-H1299 showed the highest number of adherent cells compared to COR-L105 and UP-007 (Fig. 2a–h). Adhesion of cancer cells was significantly reduced following the knockdown of *FUT4*/CD15 and the number of adherent cells were less compared to the wild type (P < 0.01) (Fig. 2a–d). A significant reduction was observed after knockdown of *FUT7*/CD15s in SEBTA-001 (2.5-fold decrease) and a 2.7-fold decrease in NCI-H1299, a 2.8-fold decrease in COR-L105 and a 2.8-fold decrease in UP-007 cells compared to the wild type (P < 0.01) (Fig. 2e–h). Following the overexpression of *FUT4*/CD15 in SEBTA-001, NCI-H1299, COR-L105 and UP-007, a significant increase in adhesion (twofold) was seen compared to the adhesion of wild type control of the same cell line (Fig. 2a–d, p < 0.01). Similar results were observed for *FUT7*/CD15s (Fig. 2e–h, p < 0.01).

**Fig. 3:**
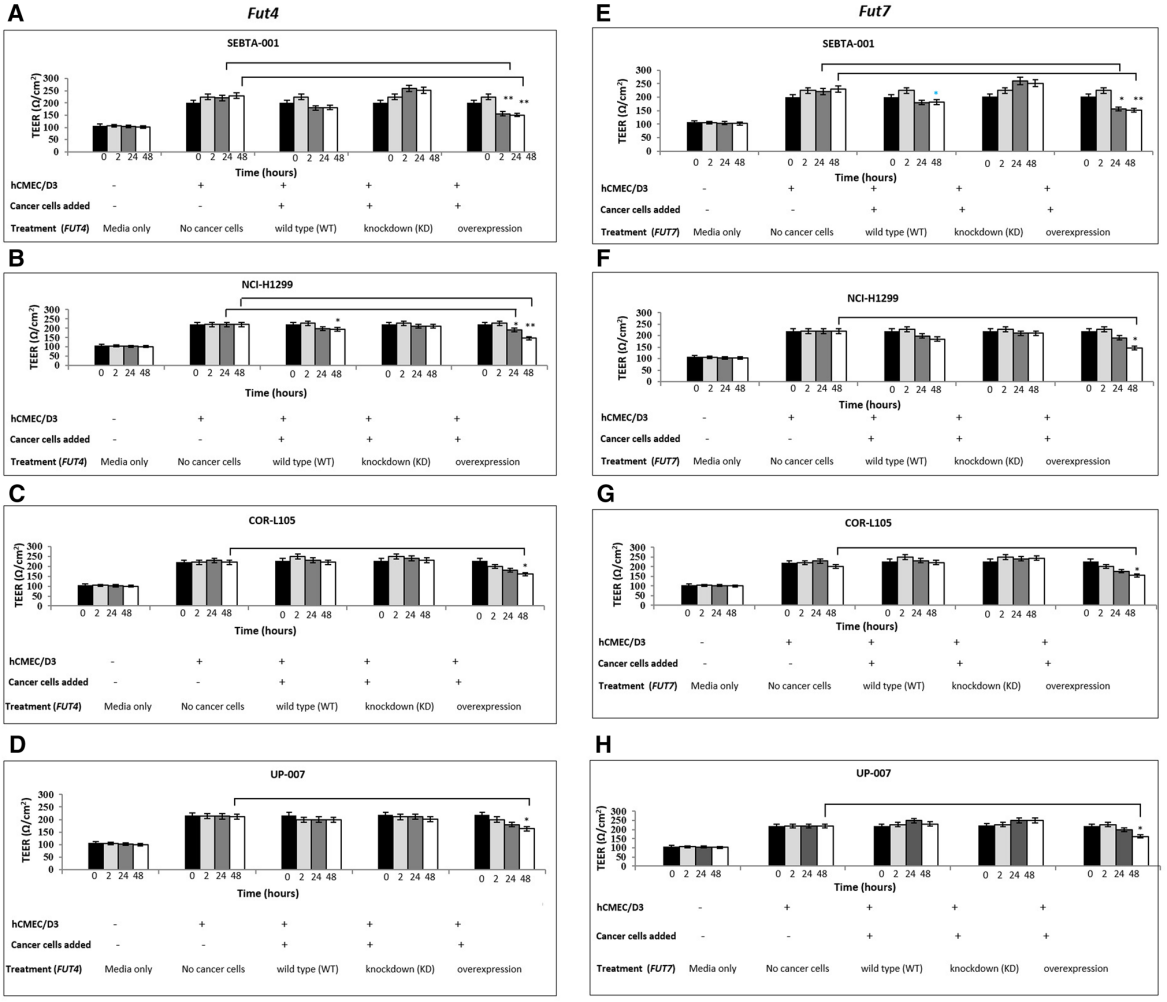
Effects of *FUT4*/CD15 and *FUT7*/CD15s modulation on intact brain endothelial monolayer assessed by EVOM™. Resistance of hCMEC/D3 monolayer in response to cancer cells following *FUT4*/CD15 (**a**–**d**) and *FUT7*/CD15s (**e**, **f**) genetic manipulation on SEBTA-001 (**a**, **e**), NCI-H1299 (**b**, **f**), COR-L105 (**c**, **g**) and UP-007 (**d**, **h**). TEER values decreased when cell lines (except for UP-007) were transfected with *FUT4/*CD15 were placed on an established endothelial cell layer. Similar data were observed for cells transfected with *FUT7*/CD15s.The black bars represents the resistance of endothelial monolayer before adding cancer cells, light-grey bars to TEER values 2 h post-addition of cancer cells, dark-grey bars to TEER values 24-h post-addition of cancer cells and white bars to TEER values 48-h post addition of applying cancer cells. **a**–**c**; *p < 0.01, **p < 0.05. N = 3

We next measured changes in transendothelial electrical resistance (TEER) using three independent technologies [16]. The EVOM™ technology, often referred to as the ‘chop-stick’ method was used to collect data every 2 h for 48 h. When wildtype SEBTA-001 and NCI-H1299 cells were added to an intact hCMEC/D3 monolayer, a decrease in resistance was observed over time and at the 48 h time point, this was significant (Fig. 3a, b, e, f; p < 0.01), whereas wildtype COR-L105 and UP-007 cells did not cause a change in resistance over time (Fig. 3c, d, g, h). Knock-down experiments targeting either *FUT4* or *FUT7* in SEBTA-001 and NCI-H1299 cells attenuated the changes in resistance seen with respective wildtype cells (Fig. 3a, b, e, f).

Enhanced expression of *FUT4* or *FUT7* in all cell lines resulted in a significant decrease in TEER values at 48 h (Fig. 3a–h; p < 0.05). Although there appears to be a trend towards a decrease in TEER values in UP-007 cells transfected with *FUT4*, UP-007 cells transfected with *FUT7*, did result in a significant decrease in TEER values at the 48 h time point (p < 0.05; Fig. 3d vs. h).

**Fig. 4:**
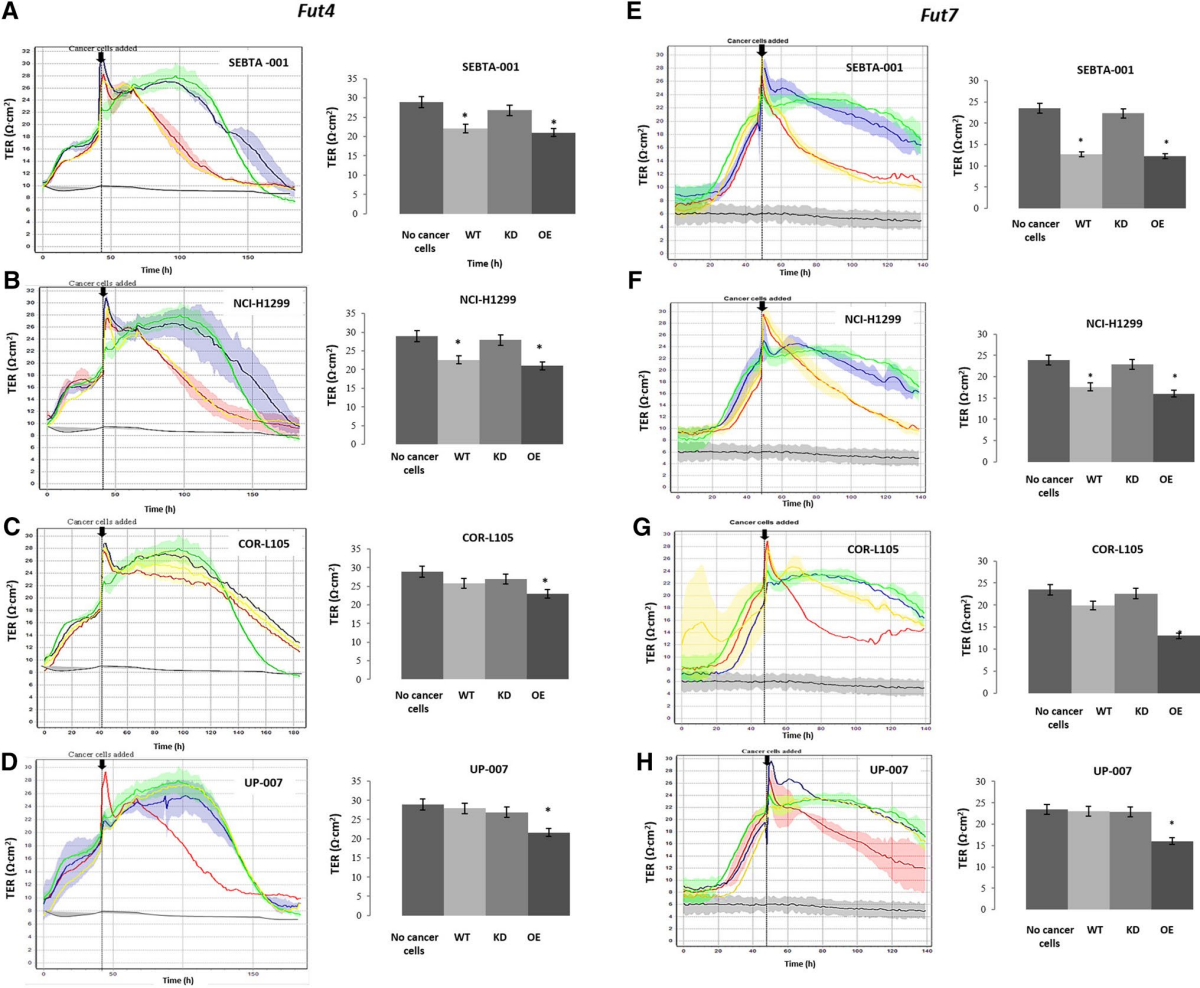
Effects of *FUT4*/CD15 and *FUT7*/CD15s modulation in cells on brain endothelial monolayer measured in real-time. Representative histograms depicting the changes in TEER values, pre and post addition of cancer cells using the automated system (CellZscope®). The green line represents endothelial cells (control) with no addition of cancer cells, red line represents *FUT4*/CD15 overexpressor cells (**a**–**d**) or *FUT7*/CD15s (**e**–**h**), blue line represents *FUT4*/CD15 shRNA transfected cells (**a**–**d**) or *FUT7*/CD15s shRNA cells (**e**–**h**), yellow line representing the wild type and black line representing the blank. Bar chart represents quantified TER values (*P < 0.05), N = 3. *WT* wild type, *KO* knockdown, *OE* overexpression

The CellZscope® methodology offers a real-time acquisition of resistance data. Addition of wildtype SEBTA-001 and NCI-H1299 to an intact monolayer of hCMEC/D3 cells resulted in a significant decrease in Transendothelial Resistance TER values over time compared to the non-metastatic cell lines, COR-L105 and UP-007 (Fig. 4a–h; p < 0.05. Bar graphs represent 24 h). The silencing of either *FUT4* or *FUT7* resulted in the decrease in the ability of SEBTA-001 and NCI-H1299 to affect hCMEC/D3 monolayer resistance (Fig. 4a–h). The overexpression of either *FUT4* or *FUT7* in cell lines tested (except for UP-007 transfected with *FUT4*), when plated onto a stable hCMEC/D3 monolayer, resulted in a significant reduction in TER values over time (Fig. 4a–h; p < 0.05).

**Fig. 5:**
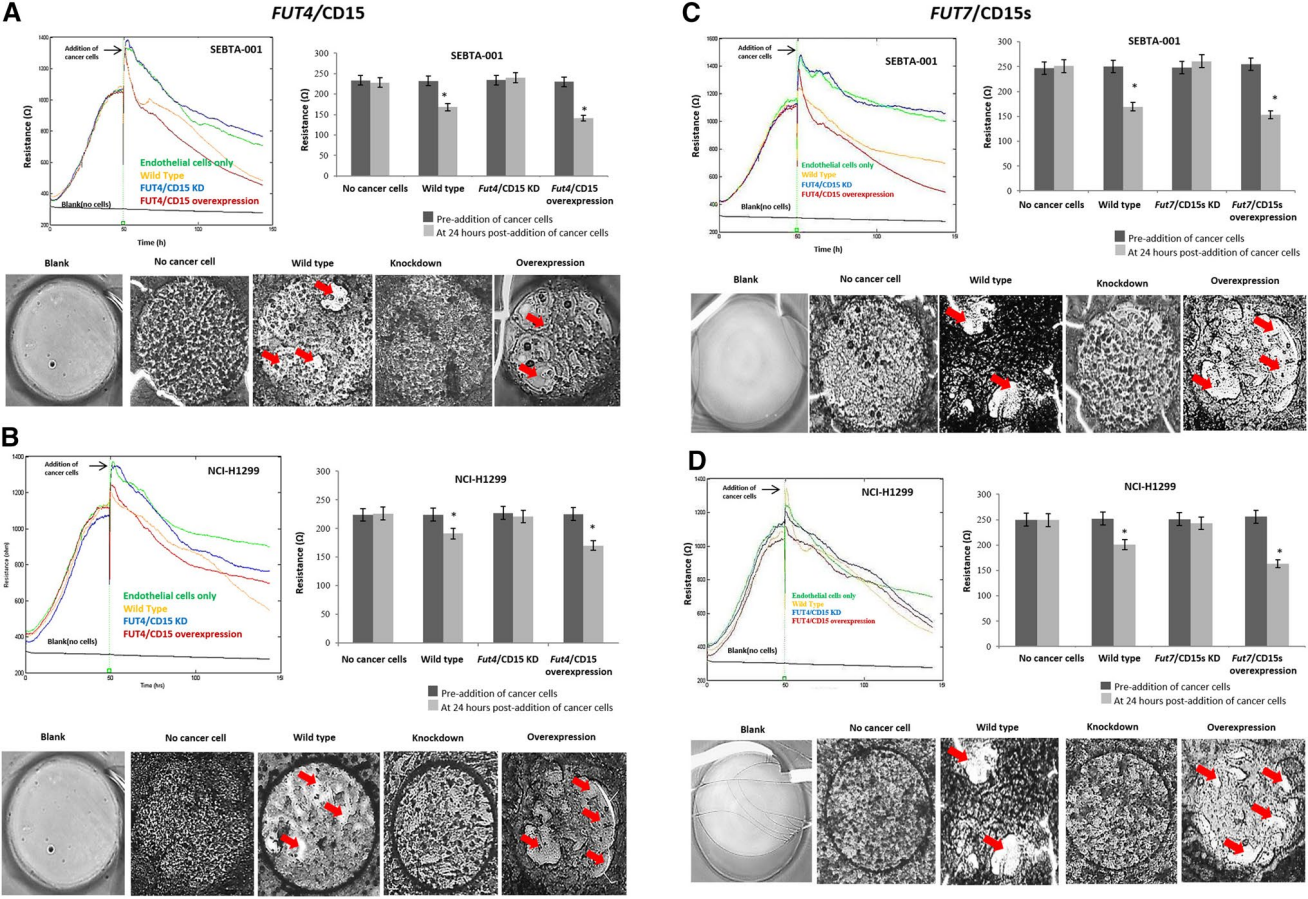
*FUT4* and *FUT7* knockdown in metastatic cancer cells prevents disruption of intact brain endothelial monolayer. *FUT4* in SEBTA-001 (**a**) and NCI-H1299 (**b**). *FUT7* in SEBTA-001 (**c**) and NCI-H1299 (**d**). **a**–**d** Representative histograms showing resistance of endothelial barrier in real time, pre and post addition of cancer cells measured by (ECIS™). Green line represents control (no cancer cells), Red line represents *FUT4*/CD15 (**a**,** b**) or *FUT7*/CD15s (**c**, **d**) overexpressing cancer cells, blue line represents knockdown cell lines *FUT4*/CD15 (**a**,** b**) or *FUT7*/CD15s (**c**, **d**), yellow line represents wildtype cancer cells and black line represents blank no cells. (**a**–**d**) Bar charts represent quantified resistance values at 24-h pre-post-addition of cancer cells (*P < 0.05). Lower panel **a**–**d**) Representative microscopic images demonstrating the changes of integrity in brain endothelial monolayer, at time point of 24 h post-addition of cancer cells. Red arrows point to areas of endothelial cell monolayer disruption. Images were obtained using a phase contrast microscope (× 4). All studies N = 3

ECIS™ results were consistent with EVOM™ and CellZscope® findings, and a strong association was noted between the expression of *FUT4*/CD15 and *FUT7*/CD15s and the decrease in the resistance of the brain endothelial monolayer. ECIS graphs and microscopic images demonstrate the changes in resistance of the endothelial cell monolayer, pre- and 24 h-post-addition of cancer cells. Addition of wildtype SEBTA-001 and NCI-H1299 cells led to a significant reduction in resistance from 232 to 168 Ω and from 224 to 190 Ω (p < 0.05) respectively (Fig. 5a–d). In support of endothelial layer disruption, microscopic images show wide gaps in the endothelial monolayer barrier (arrows; Fig. 5). Knockdown of CD15 and CD15s in SEBTA-001 and NCI-H1299 did not result in significant changes to resistance. In addition, images of monolayer show a well-structured endothelial monolayer (Fig. 5d). Similar to the other methods used for measuring BBB integrity, both CD15 and CD15s overexpressing cells led to dramatically reduction in resistance (Fig. 5a–d; Supplemental Fig. 1a–d).

## Discussion

CD15 and CD15s, well characterized for their involvement in the homing process of leukocytes, are correlated with cancer progression and metastasis in non-CNS cancers [17–19]. In previous studies, we showed that CD15 and CD15s are involved in the adhesion of cancer cells to brain endothelium via CD62E interaction [11, 12]. Here, we wanted to further investigate the role of CD15 and CD15s on the potential transmigration of cancer cells across an intact brain endothelial monolayer by modulating CD15 and CD15s expression by targeting the *FUT4* and *FUT7* genes.

CD15 and CD15s biosynthesis is regulated by fucosyltransferase enzymes (alpha-1,3 FUTs); FUT3,-4,-5,-6,-7 and -9, which are spatial and temporally regulated and which expression appears to be cell/tissue specific [20–23]. Overexpression of *FUT4* leads to the increase in CD15 expression and correlates with metastasis in colorectal cancer [24]. Conversely, *FUT4* knockdown is reported to reduce the expression of CD15 in promyelocytes and monocytes [25]. *FUT4* and *FUT7* knockdown was shown to significantly reduce the trafficking of leukocytes and lymphocytes [26]. FUT overexpression in general has been correlated with poor prognosis and metastasis in prostate cancer [27–29] as well as lung cancer [30].

Overexpression of *FUT4*/CD15 led to an increase in endothelial cell adhesion in all studied cancer cells and *FUT4*/CD15 knockdown resulted in decreased adhesion. These findings are in agreement with that of Yang et al. [31] where overexpression of *FUT4* correlated with high metastatic potential in breast cancer cells and implicated in adhesion and invasion. Knockdown of *FUT4*/CD15 and *FUT7*/CD15s significantly reduced the number of adherent metastatic and primary lung cancer cells as well as GBM cells to brain endothelium.

The findings related to the GBM cell line are very interesting. GBM rarely metastasises to other organs [32]. Martin et al. in 1995 [32] suggested that the absence in CD15 expression in GBM cells may contribute to the failure of GBM cells to metastasise due to the inability for glioma cells to attach to brain endothelial cells. The expression dynamics of CD15/CD15s in GBM could help to explain the rarity of GBM extraneural metastasis. Could CD15/CD15s expression dynamics be a mechanism in which secondary lesions appear remote from the initial presentation site? GBM cells which have exited and entered the peripheral circulation may at least in theory, re-enter the brain to set up a second lesion site. This re-entry phenomenon if proven true may offer a second possibility underlying the observation of multiple GBM lesions currently believed to be due to invasion of GBM rogue cells. At this stage, this theory needs to be further investigated.

While our data suggests a role of CD15/s in NSCLC metastasis to brain, there remain questions into mechanisms for trans-endothelial transmigration of cancer cells. A likely scenario involves a bi-directional initiation of signalling cascades leading to the orchestration of processes such as the release of enzymes responsible for the breakdown of basal lamina for example. All of which underpin migration and invasion into the brain. This is followed by colonisation of the brain by interplay between tumour cells and the brain micro-environment.

Recently Zhao et al. [33], identified a prognostic signature from a comprehensive bioinformatic analyses of 500 lung adenocarcinoma samples. One of the 20 genes highlighted, *FUT4*, was found to be one of the most significant genes associated with poor survival. It would be interesting to determine if the *FUT* genes are associated with brain metastases. Another clinical aspect of our studies is the potential to use these epitopes as therapeutic targets to prevent cerebral metastases of NSCLC by blocking adhesion to brain endothelial cells. While there are several temporal doorways for therapeutic targeting of metastatic spread, from intravasation to development of secondary lesions in the brain, it is generally agreed that once in the brain, the lesion is protected from the full benefits of treatment due to the blood brain barrier. With the advances in biomarkers and in liquid biopsies of circulating cancer cells, the possible use of these epitopes as biomarkers for metastasis merits further study.

## Conclusions

This study sheds light on two fucosyltransferases (FUT4 and 7) and the role of CD15 and CD15s expression in adhesion to and disruption of cerebral endothelial cell monolayers. Our data demonstrate that blocking of CD15 or CD15s expression weakens lung cancer cell adhesion and impedes the disruption of an in vitro model of a BBB model. Surprisingly, over-expression of these epitopes by inducing *FUT4* or *FUT7* in cell lines not known to be ‘metastatic’ become ‘metastatic-like’ in regards to disruption of the BBB. While exciting, future in vivo studies are needed to confirm our in vitro observations.

## Electronic supplementary material

Below is the link to the electronic supplementary material.

Supplementary material 1 (EPS 1304 KB)
